# The Synergistic Antidepressant Effect: Compatibility of Alkaloids with Saponins from *Ziziphi Spinosae Semen*

**DOI:** 10.1155/2022/5755980

**Published:** 2022-04-16

**Authors:** Lu Li, Wei Song, Qianqian Chang, Yan Sun, Demin Fang, Wei Qiao

**Affiliations:** ^1^Tianjin Hospital, Tianjin 300211, China; ^2^Tianjin Key Laboratory on Technologies Enabling Development of Clinical Therapeutics and Diagnostics, School of Pharmacy, Tianjin Medical University, Tianjin 300070, China

## Abstract

*Context. Ziziphi Spinosae Semen* (ZSS) is a well-known Chinese herbal medicine used in the treatment of depression and anxiety in China. ZSS contains several active components, such as alkaloids, saponins, and flavonoids. *Objective*. This study aimed to explore the synergistic effect of alkaloids and saponins from ZSS in alleviating depression in a mouse model. *Materials and Methods*. Modeling depression with chronic unpredictable stimuli. Pharmacodynamic methods (tail suspension test and forced swimming test) were used to evaluate the antidepressant effects of alkaloids, saponins, and combinations thereof from ZSS. The mechanisms underlying the effect were examined by measuring the levels of monoamine neurotransmitters in the hippocampus and frontal cortex of mice. *Results*. Compared with the model group, alkaloids therapy (AZSS), saponins therapy (SZSS), and combination therapy significantly reduced the immobility time in behavioral tests (*P* < 0.05). The contents of noradrenaline (NE), dopamine (DA), and serotonin (5-HT) in the hippocampus and frontal cortex of depressed mice were increased in the drug treatment groups, especially in the combination group, which increased by 14.65%, 87.72%, 33.44%, 25.64%, 25.39%, and 70.78%, respectively. Several groups showed better results (*P* < 0.05), especially the combination of alkaloids and saponins. *Discussion and Conclusion*. The saponins and alkaloids from ZSS exhibited a synergistic effect in improving the behavior of depressed mice. More importantly, the combination of alkaloids (15 mg·kg^−1^) and saponins (110 mg·kg^−1^) was effective in alleviating depression in mice, especially in terms of changing the level of DA in the hippocampus.

## 1. Introduction

Depression is a common psychiatric disorder. It is often associated with changes in appetite and sleeping patterns, agitation or retardation, decreased energy, feelings of worthlessness or guilt, impairment in concentration, and recurrent thoughts of death or suicide [1]. The incidence of depression in adults is 5.0% in the world and 3.02% in China [2, 3]. The World Health Organization (WHO) has listed depression as a leading cause of disability worldwide and a major contributor to the overall global burden of disease (https://www.who.int/news-room/fact-sheets/detail/depression). The pathological mechanism of depression is very complex, and there is no uniformity on the mechanism to date. Currently existing antidepressants mainly include selective serotonin uptake inhibitors, tricyclics, and others. These types of antidepressants, however, suffer from severe drawbacks such as single target, adverse reactions, high drug price, and high recurrence rate. Herbal medicines are commonly employed to treat mental disorders because of their ability to act on different mechanisms [4, 5]. Hence, finding out multitarget antidepressant herbal medicines with a low level of toxicity will be of high significance.

In the theories of traditional Chinese medicine (TCM), depression belongs to “yuzheng,” which is closely related to the stagnation of “qi” in the liver. The “qi,” in TCM, is applied to describe one pattern of bodily disharmony [6]. Thus, the main treatment methods for depression are soothing the liver and regulating “qi,” together with replenishing “qi” to invigorate the spleen, activating blood to resolve stagnation, and reducing phlegm to resolve masses [6]. As a Chinese herbal medicine with depression-resolving and spirit-tranquilizing effects and a low level of toxicity, *Ziziphi Spinosae Semen* (ZSS) is the dried seed of *Ziziphus jujube* var. *spinosa* (Bunge) Hu ex H.F. Chow. ZSS exhibits pharmacological effects of nourishing the liver, arresting sweating, calming the heart, and promoting fluid production [7]. Our previous research along with recent reports have supported that saponins and alkaloids found in ZSS display sedative, hypnotic, and antidepressant activities [8–10]. However, no literature has explored whether saponins and alkaloids from ZSS have a synergistic antidepressant effect.

Research has shown that medicinal plants can affect the nervous system and exert antidepressant effects in various ways, including regulation of serotonin, noradrenaline, dopamine, and inflammatory mediators in synapses [11]. In this study, we utilized a mouse model of depression to investigate the effects of different components of ZSS on depressed mice's behavior and levels of brain transmitters, thereby exploring whether there is a synergistic-effect relationship among the effective components of ZSS and their possible mechanisms of action in alleviating depression.

## 2. Materials and Methods

### 2.1. Animals

Male ICR mice weighing 18–20 g were used in this study (Animal Center, Institute of Radiological Medical, Chinese Academy of Medical Sciences, Certificate No. SCKX-2009-0004). Ten mice were kept in one cage (dimension of the cage: 440 mm × 270 mm × 178 mm) under a standard condition (12-hour light/dark cycle; lights on at 7 : 00 am; temperature: 22 ± 2°C; humidity: 65%–70%) with free access to food and water. The mice were adapted to the laboratory for at least three days before experiments. All the experiments were conducted between 9 : 00 am and 3 : 00 pm and performed in accordance with the NIH (2011) Guide for Care and Use of Laboratory Animals.

### 2.2. Reagents

Dopamine (DA) was purchased from Tokyo Chemical Industry Co. Ltd. (Tokyo, Japan). 5-Hydroxytryptophan (5-HTP) was obtained from Fluka AG Chemical Co. (Buchs, Switzerland). Noradrenaline (NE) was purchased from the National Institutes for Food and Drug Control (Beijing, China). Venlafaxine was supplied by Wyeth Medica Ireland (Kildare, Ireland). All the other reagents used in the study were of analytical grade.

### 2.3. Experimental Materials and Preparation of Standardized Alkaloid (AZSS) and Saponin (SZSS) Extracts from ZSS

**Figure 1:**
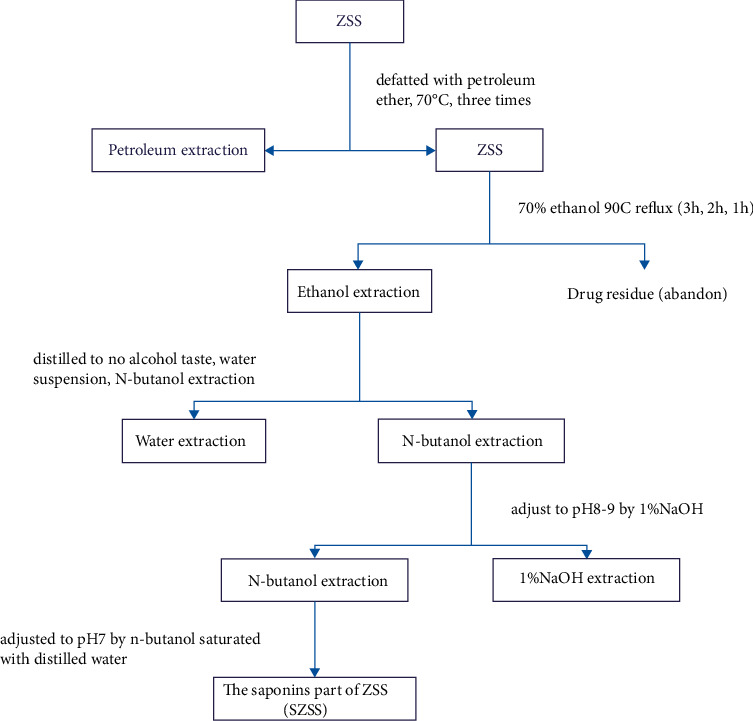
The extraction process of total saponins from ZSS.

**Figure 2:**
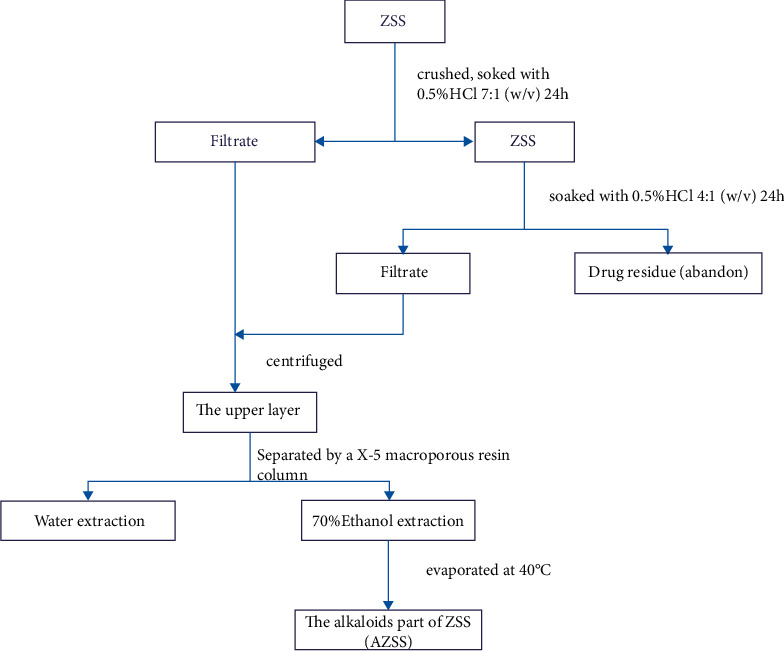
The extraction process of total alkaloids from ZSS.

ZSS materials (Lot: 20190860), which were produced in Hebei Province and picked in autumn, were purchased from Bozhou Medicinal Materials Company. The identity of ZSS was verified by Professor Ye Zhou (Tianjin Medical University, China). Saponins and alkaloids were extracted from parts of ZSS following the flow charts shown in Figures 1 and 2.

**Figure 3:**
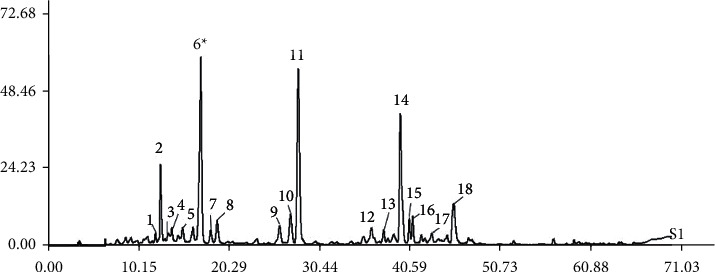
Chromatogram of the alkaloids part of ZSS analyzed by HPLC-DAD technique. (peak 6: magnoflorine).

The composition of AZSS was evaluated on an Agilent 1100 series HPLC equipped with a diode array detection (DAD). Chromatographic separation was performed using an Ameritech Accurasil C18 column (250 mm × 4.6 mm, i.d. = 5 mm). Acetonitrile (mobile phase A) and water with 0.1% phosphoric acid (mobile phase B) were applied for gradient elution. The setting of gradient elution was 0–10 min, 0–15% acetonitrile; 10–30 min, 15%–20% acetonitrile; 30–45 min, 20%–30% acetonitrile; 45–55 min, 30%–40% acetonitrile; 55–60 min, 40%–60% acetonitrile; 60–65 min, 60%–95% acetonitrile; and 65–70 min, 95%–100% acetonitrile. The wavelength used for analysis was 204 nm. According to the reference standard in the database, the main component of AZSS was determined to be magnoflorine (Figure 3).

**Figure 4:**
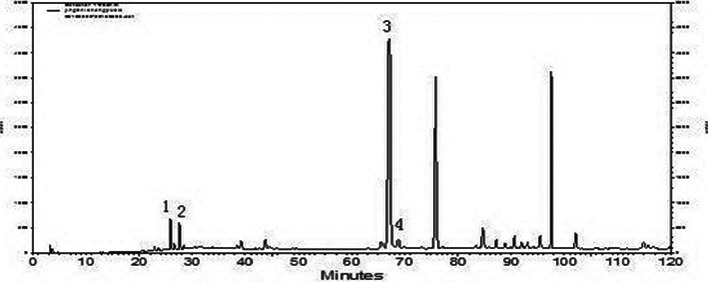
Chromatogram of the saponins part of ZSS analyzed by the HPLC-DAD technique (peak 1: jujuboside A; peak 2: jujuboside B; peak 3: betulinic acid; peak 4: betulinol).

The composition of SZSS was analyzed under the same chromatographic conditions as those used for AZSS analysis. The setting of gradient elution was 0–12 min, 22%–30% acetonitrile, 12–13 min, 30%–33% acetonitrile; 13–26 min, 33%–39% acetonitrile; 26–31 min, 39%–42% acetonitrile; 31–34 min, 42% acetonitrile; 34–40 min, 42%–63% acetonitrile; 40–56 min, 63%–77.8% acetonitrile; 56–62 min, 77.8% acetonitrile; 62–66 min, 77.8%–78.2% acetonitrile; 66–85 min, 78.2%–100% acetonitrile; and 85–90 min, 100% acetonitrile. Comparative analysis with chromatographic references showed that the main components of SZSS were jujuboside A, jujuboside B, betulinic acid, and betulinol (Figure 4).

### 2.4. Drug Administration

The mice were randomly divided into 6 groups (*n* = 10 per group) to establish one normal control and 5 experimental groups (Table 1). All animals, except those in the normal control group, were orally administered with different drug regimens shown in Table 1 once a day for 21 d between 9 : 00 am and 2 : 00 pm. The dosages of the experimental groups were obtained through preliminary study.

### 2.5. Establishment of the Mouse Model of Chronic Unpredicted Mild Stress (CUMS)-Induced Depression

**Figure 5:**
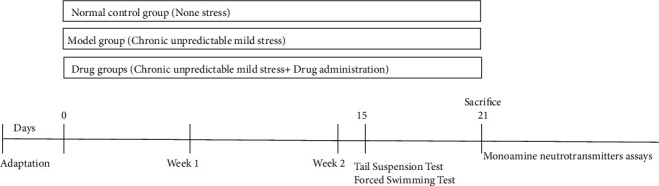
Animal experiment process.

All the mice in the 5 experimental groups were subjected to CUMS. A total of 5 stress stimulations were randomly applied to those mice, including ultrasound exposure for 30 s, tail nipping for 1 min, forced swimming in cold water (10°C) for 5 min, forced swimming in hot water (45°C) for 5 min, cage tilting at 45° for 12 h, forced staying in damp sawdust for 12 h, food deprivation for 12 h, water deprivation for 12 h, sleep deprivation, forced staying in a no-sawdust environment for 12 h, and reversal of the light/dark cycle for 24 h. Two stress stimulations were randomly selected and applied on the mice once a day for three weeks. We ensured that the same stress stimulation was not applied for two consecutive days and that one stress stimulation was applied on mice in each group for an average of 4 times before drug administration. In addition, all these stress stimulations were carried out in the breeding room of another lab [12, 13]. The specific experiment flow chart is shown in Figure 5.

### 2.6. Behavioral Studies

Behavioral tests, including tail suspension test (TST) and forced swimming test (FST), were carried out on the 15th and 17th days of stimulation, as described in our previous study [14].

### 2.7. Analysis of the Levels of Monoamine Neurotransmitters

Half an hour after the last drug administration, the animals were sacrificed by decapitation to determine the levels of noradrenaline (NE), dopamine (DA), and serotonin (5-HT). The frontal cortex and hippocampus were rapidly isolated from mice placed on an ice-cold dissection board, weighed, and stored at −80°C.

The levels of NE, DA, and 5-HT were determined based on the method described in previous studies with slight modifications [15, 16]. In brief, the brain tissue was homogenized in acidified n-butanol, shaken for 5 s, and then centrifuged at 999 *g* at 4°C for 5 min. The supernatant was mixed with *n*-heptane and 0.1 M HCl, shaken for 10 s, and then centrifuged at 999 *g* at 4°C for 5 min. The aqueous phase was obtained to determine the levels of NE, DA, and 5-HT. Specifically, for determination of NE and DA levels, 0.5 mL of the aqueous phase was transferred into test tubes and added with 1.7 mL of phosphate buffer (pH 7.2) and a 0.4 mL solution containing 0.1 M disodium ethyleneaminetetraacetic acid and 0.1 M iodine. After incubation for 2 min, 0.5 mL of 0.2 M alkaline sodium sulfite was added into the mixture, followed by incubation for another 2 min. In the end, 0.5 mL of 6 M acetic acid was added into the tubes. The tubes were placed in a boiling water bath for 2 min and then cooled in water to room temperature. The excitation and emission wavelengths for NE are 284 nm and 480 nm, respectively. In order to determine the level of DA, samples were heated for another 2 min and then cooled again. The excitation and emission wavelengths for DA are 320 nm and 369 nm, respectively. For reagent blank and sample blank, only the alkaline sodium sulfite solution was added after the iodine solution.

**Figure 6:**
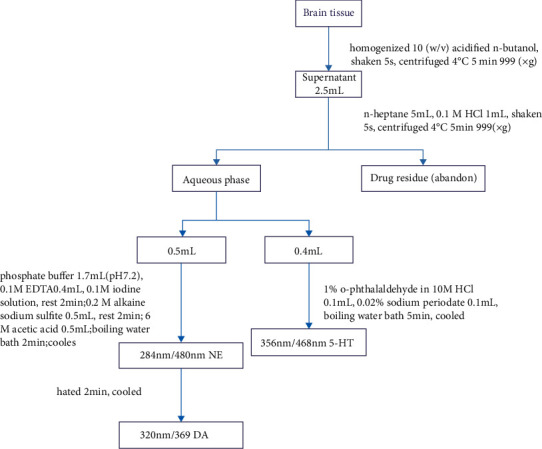
Flow chart for determination of monoamine neurotransmitters in the mouse brain.

For determination of 5-HT level, 0.4 mL of the aqueous phase was transferred to a test tube, followed by the addition of 0.1 mL of 1% *o*-phthalaldehyde in 10 M HCl and 0.1 mL of 0.02% sodium periodate. The test tube was placed in a boiling water bath, boiled for 10 min, and cooled down in the water. The excitation and emission wavelengths are 356 and 468 nm, respectively, for 5-HT. The blank control sample was prepared with 10 M HCl instead of the aqueous phase (Figure 6). The levels of NE, DA, and 5-HT were calculated according to the respective standards and expressed as ng/gram of the brain tissue.

(1) $$\begin{array}{c} \mathrm{Content}\left(\mathrm{ng\cdot}g^{\text{-1 }}\right)=\frac{\left(\text{sample tube fluorescence readings - tissue blank fluorescence readings}\right)}{\left(\text{standard tube fluorescence readings - reagent blank fluorescence readings}\right)\;}\times \left(\text{standard concentration}\right)\times \left(\text{standard volume}\right)\;\times \;\frac{3}{2\mathrm{.5}}\;\times \;\frac{1}{\text{weight of brain tissue}}\mathrm{\times 1000}. \end{array}$$

### 2.8. Statistical Analysis

All data are presented as mean ± standard error of mean (SEM). Intergroup differences were assessed for significance using one-way analysis of variance (ANOVA), followed by Dunnett's test. *P* value < 0.05 was considered statistically significant.

## 3. Results

### 3.1. The Effects of Each ZSS Group on Behavioral Parameters

**Figure 7:**
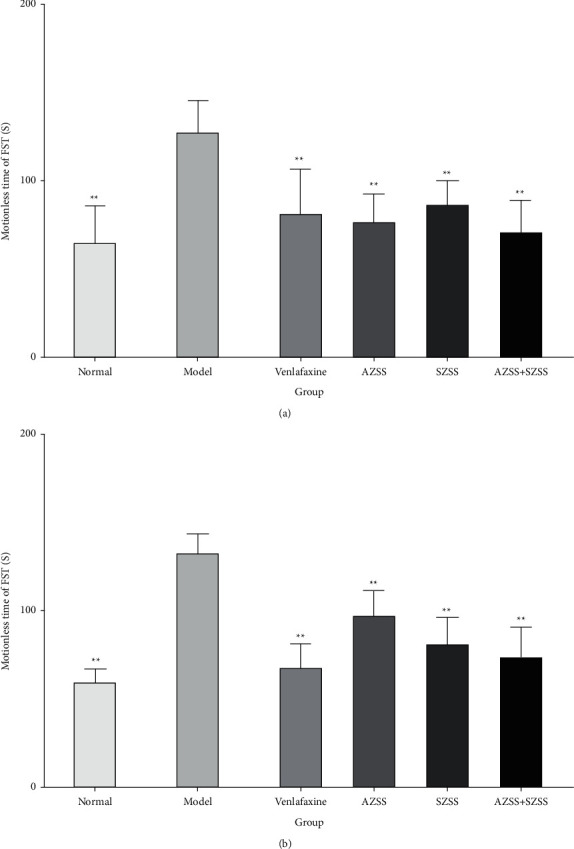
The effects of each ZSS group on the stationary time of TST (a) and FST (b) in chronic unpredicted depressed mice. Mice were administered distilled water, venlafaxine (9.38 mg·kg^−1^), AZSS (15 mg·kg^−1^), SZSS (110 mg·kg^−1^), and AZSS + SZSS (15 mg·kg^−1^ + 110 mg·kg^−1^). Values are presented as mean ± SEM (*n* = 10). ^*∗∗*^*P* < 0.01 vs the model group.

Behavioral tests were performed after the two-week treatment with CUMS (Figure 7). The CUMS resulted in a significantly prolonged immobility time of mice in the TST&FST when compared with the mice of the normal control group ( *P* < 0.01). The mice treated with ZSS exhibited a significantly decreased immobility time compared with the model group, which suggested that ZSS could alleviate CUMS-induced depression-like behaviors in the TST&FST tests ( *P* < 0.01).

### 3.2. The Effects of ZSS on Monoamine Neurotransmitter Levels in Different Brain Regions of the Depressed Mice

#### 3.2.1. Hippocampus

**Figure 8:**
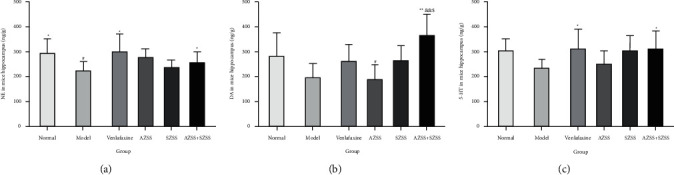
The effects of each ZSS group on the levels of NE (a), DA (b), and 5-HT (c) in the hippocampus of the chronic unpredicted depressed mice. Mice were administered distilled water, venlafaxine (9.38 mg·kg^−1^), AZSS (15 mg·kg^−1^), SZSS (110 mg·kg^−1^), and AZSS + SZSS (15 mg·kg^−1^ + 110 mg·kg^−1^). Values are presented as mean ± SEM (*n* = 10). ^*∗*^*P* < 0.05 and ^*∗∗*^*P* < 0.01 vs the model group, ^#^*P* < 0.05 vs the normal control group, ^&&^*P* < 0.01 vs AZSS, and ^$^*P* < 0.05 vs SZSS.

Compared with the model group, the levels of NE, DA, and 5-HT in the mice hippocampus were significantly increased in drug treatment groups (Figure 8), especially the AZSS + SZSS group (*P* < 0.05). The levels in the model group showed an apparent decrease relative to that in the normal control group, but only the NE level had statistical differences (*P* < 0.05).

The DA level in the AZSS + SZSS group showed a noticeable increase (*P* < 0.01), compared with the model group. It is worth noting that the AZSS + SZSS group demonstrated a more significant increase in DA level than the AZSS group (*P* < 0.01) and the SZSS group (*P* < 0.05).

#### 3.2.2. Frontal Cortex

**Figure 9:**
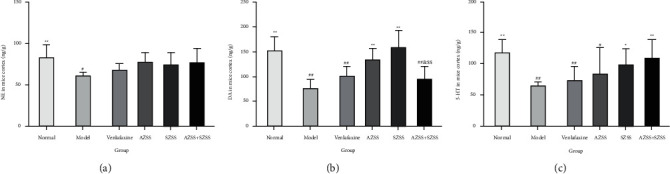
The effects of each ZSS group on the levels of NE (a), DA (b), and 5-HT (c) in the frontal cortex of the chronic unpredicted depressed mice. Mice were administered distilled water, venlafaxine (9.38 mg·kg^−1^), AZSS (15 mg·kg^−1^), SZSS (110 mg·kg^−1^), and AZSS + SZSS (15 mg·kg^−1^ + 110 mg·kg^−1^). Values are presented as mean ± SEM (*n* = 10). ^*∗*^*P* < 0.05 and ^*∗∗*^*P* < 0.01 vs the model group, ^#^*P* < 0.05 and ^##^*P* < 0.01 vs the normal control group, ^&^*P* < 0.05 vs AZSS, and ^$$^*P* < 0.01 vs SZSS.

The contents of NE, DA, and 5-HT in the frontal cortex of mice from different groups are shown in Figure 9. Compared with the normal control group, the levels of monoamine neurotransmitters in the model group had decreased sharply (*P* < 0.05), especially in the DA and 5-HT level (*P* < 0.01).

The measured contents of monoamine neurotransmitter in drug treatment groups were higher than that in the model group. Specifically, the DA levels of AZSS and SZSS were more significantly different from the model group (*P* < 0.01). Moreover, the SZSS group (*P* < 0.05) and the AZSS + SZSS group (*P* < 0.01) exhibit statistically significant differences in 5-HT levels relative to the model group.

In addition, AZSS and SZSS did not show a synergistic effect in the DA level, and it was significantly lower in the compatibility group than the AZSS group (*P* < 0.05) and the SZSS group (*P* < 0.01).

## 4. Discussion

Initial pharmacological studies on the components of ZSS suggested that the herbal medicine has antidepressant activities and contains both AZSS and SZSS, but the mechanism underlying its antidepressant effect is unclear [9, 10]. More recent work on herbal formulae containing ZSS has demonstrated that the underlying mechanism may be related to the increase in 5-HT in peripheral blood and 5-hydroxyindoleacetic acid (5-HIAA) in the brain [17]. Our data linking AZSS and SZSS to the antidepressant activity of ZSS indicate that monoamine transmitters in the brain are also implicated in this mechanism. Inspired by the presence of Chinese herbal compounds, we combined AZSS and SZSS to form a formula, which could, in principle, maximize the efficacy of ZSS; that is, the AZSS + SZSS treatment may be more effective than AZSS or SZSS alone. We therefore investigated the possible synergistic effect between AZSS and SZSS in depression treatment. Our TST and FST data established that AZSS and SZSS had a certain synergistic effect in improving the behavior of depressed mice, especially the treatment of AZSS + SZSS 15 mg·kg^−1^ + 110 mg·kg^−1^, which was most effective in terms of changing the DA level in the hippocampus of depressed mice.

To better investigate the antidepressant mechanisms of ZSS, we established a CUMS-induced mouse depression model, which has been widely used in simulating human depression [18, 19]. There is growing evidence that the depletion of monoamine transmitters in the central nervous system is involved in the pathological mechanism underlying the formation of CUMS-induced depression [20]. Based on this, we speculated that AZSS and SZSS improved depression-like behavior in the CUMS-induced depression mouse model by regulating the levels of monoamine transmitters in the brain. In recent years, serum 5-HT level has been shown to play an important role in directly indicating brain 5-HT level [17]. Therefore, this method should have been used in this study to monitor the dynamic changes of 5-HT levels in CUMS-induced depressed mice treated with ZSS throughout the entire experiment. This can be considered as a limitation of this study.

The synergistic effect between AZSS and SZSS has also been demonstrated by the DA level changes in the brains of CUMS-induced depressed mice: AZSS + SZSS exhibited a more significant effect than AZSS or SZSS alone. As a dominant transmitter in the extrapyramidal system of the brain, dopamine is a precursor for epinephrine and norepinephrine and plays an important role in regulating behavior [21]. We showed that AZSS + SZSS had opposite effects in altering DA levels in the hippocampus versus in the cortex of depressed mice. Further in-depth studies, based on the work reported here, are needed to explore the regulation mechanism of dopamine transporters by AZSS and SZSS.

## Figures and Tables

**Table 1:** Drug administration.

No.	Drug administration	Equal to ZSS (g·kg^−1^)
1	Normal control (none)	
2	Model (distilled water)	
3	Venlafaxine^*∗*^(9.38 mg kg^−1^)	
4	AZSS (15 mg kg^−1^)	0.6
5	SZSS (110 mg kg^−1^)	3.8
6	AZSS + SZSS (15 mg kg^−1^ + 110 mg kg^−1^)	4.4

^*∗*^Venlafaxine was used as the positive control.

## Data Availability

No data were used to support this study.

## Abbreviations

ZSS: *Ziziphi Spinosae Semen*
AZSS: Alkaloid part of ZSS
SZSS: Saponins part of ZSS
TST: Tail suspension test
FST: Forced swimming test
DA: Dopamine
NE: Noradrenaline
5-HT: Serotonin
5-HTP: 5-Hydroxytryptophan
HPLC-DAD: High-performance liquid chromatography-Diode array detection.
